# Characterization of Resistance in Gram-Negative Urinary Isolates Using Existing and Novel Indicators of Clinical Relevance: A 10-Year Data Analysis

**DOI:** 10.3390/life10020016

**Published:** 2020-02-11

**Authors:** Márió Gajdács, Zoltán Bátori, Marianna Ábrók, Andrea Lázár, Katalin Burián

**Affiliations:** 1 Department of Pharmacodynamics and Biopharmacy, Faculty of Pharmacy, University of Szeged, Eötvös utca 6., 6720 Szeged, Hungary; 2 Department of Ecology, Faculty of Sciences, University of Szeged, Közép fasor 52., 6726 Szeged, Hungary; zbatory@gmail.com; 3 Institute of Clinical Microbiology, Faculty of Medicine, University of Szeged, Semmelweis utca 6., 6725 Szeged, Hungary; abrok.marianna@med.u-szeged.hu (M.Á.); lazar.andrea@med.u-szeged.hu (A.L.); 4 Department of Medical Microbiology and Immunobiology, Faculty of Medicine, University of Szeged, Dóm tér 10., 6720 Szeged, Hungary; burian.katalin@med.u-szeged.hu

**Keywords:** clinical microbiology, indicators, urinary tract infection, Gram-negative, drug resistance, usual drug resistance, difficult-to-treat resistance, UDR, DTR, MDR, XDR, PDR

## Abstract

Classical resistance classifications (multidrug resistance [MDR], extensive drug resistance [XDR], pan-drug resistance [PDR]) are very useful for epidemiological purposes, however, they may not correlate well with clinical outcomes, therefore, several novel classification criteria (e.g., usual drug resistance [UDR], difficult-to-treat resistance [DTR]) were introduced for Gram-negative bacteria in recent years. Microbiological and resistance data was collected for urinary tract infections (UTIs) retrospectively, corresponding to the 2008.01.01–2017.12.31. period. Isolates were classified into various resistance categories (wild type/susceptible, UDR, MDR, XDR, DTR and PDR), in addition, two new indicators (modified DTR; mDTR and mcDTR) and a predictive composite score (pMAR) were introduced. Results: n = 16,240 (76.8%) outpatient and n = 13,386 (69.3%) inpatient UTI isolates were relevant to our analysis. *Citrobacter-Enterobacter-Serratia* had the highest level of UDR isolates (88.9%), the *Proteus-Providencia-Morganella* group had the highest mDTR levels. MDR levels were highest in *Acinetobacter* spp. (9.7%) and *Proteus-Providencia-Morganella* (9.1%). XDR- and DTR-levels were higher in non-fermenters (XDR: 1.7%–4.7%. DTR: 7.3%–7.9%) than in *Enterobacterales* isolates (XDR: 0%–0.1%. DTR: 0.02%–1.5%). Conclusions: The introduction of DTR (and its’ modifications detailed in this study) to the bedside and in clinical practice will definitely lead to substantial benefits in the assessment of the significance of bacterial resistance in human therapeutics.

## 1. Introduction

The emergence and worldwide spread of antibiotic-resistant bacterial pathogens is one of the most serious concerns for modern medicine and a major public health issue, which requires participation of all the relevant stakeholders on a global scale [1,2,3,4,5]. Infections caused by drug resistant bacteria are associated with decreased quality of life (QoL) in the affected patients, increased costs for the healthcare infrastructure, the necessity to use antibiotics that are more expensive or have a disadvantageous toxicity profile and an increased mortality rate overall [6,7,8]. To make matters worse, the pipeline in the field of antibiotic discovery and the development of novel agents is plagued by a strong disincentive for drug developers, as the return-on-investment for these drugs pales in comparison to other drugs for the therapy of chronic non-communicable diseases or cancer [9,10]. Although in recent years, so-called public-private partnerships (including the *10 × 20 Initiative* of the US Food and Drug Administration and the *New Drugs 4 Bad Bugs [ND4BB]* programme of the European Medicines Agency) have tried to make the field of antimicrobial discovery an attractive field of investment for pharmaceutical companies, it is a far cry from the golden age of antibiotic discovery (1960–1980), when new drugs continuously appeared on the market [11,12,13]. Some reports have gone as far as to suggesting that mortality due to drug resistant pathogens will be one of the leading causes of death by 2050, surpassing the mortality rate of malignant illnesses [14].

If overall mortality and their economic impact is taken into consideration, the group of “ESKAPE” pathogens, namely E: *Enterococcus faecium*, S: *Staphylococcus aureus* or recently *Stenotrophomonas maltophilia*, K: *Klebsiella pneumoniae* or recently C: *Clostridioides difficile*, A: *Acinetobacter baumannii*, P: *Pseudomonas aeruginosa*, E: *Enterobacter* spp., or recently *Enterobacteriaceae*) present the most clinical challenges [15,16]. A plethora of resistance mechanisms has been described in various bacterial species; some of these resistance mechanisms are plasmid-mediated, which allows for their widespread dissemination and outbreak-formation (particularly in Gram-negative bacteria; e.g., transmission of carbapenemase genes), while some bacteria possess intrinsic resistance mechanisms present in all species (e.g., resistance to tetracyclines, nitrofurantoin and polymyxin B in *Proteus*, *Providencia* and *Morganella* species) [17,18,19,20]. During susceptibility-testing and choosing the appropriate therapy, both clinicians and clinical microbiologists need to be aware of intrinsic resistance and local developments in acquired resistance levels [21]. 

As the clinical problem of bacterial drug resistance has emerged, several methods were developed to classify bacterial species into resistance categories; however, due to differences in these classifications, notable inconsistencies occurred in the reporting of epidemiological and clinical results [22]. The first global consensus criteria for the classification of drug resistance levels in bacteria were outlined by the collaborative efforts of the US Centers for Disease Control and Prevention (CDC) and the European Centre for Disease Control and Prevention (ECDC), namely the definitions for multidrug resistance (MDR), extensive drug resistance (XDR) and pan-drug resistance (PDR) [23]. These resistance categories are useful in reporting epidemiological data for the assessment of the resistance situation is a given geographical region. However, this categorization of resistance does not take into account the differences in the clinical utility and pharmacological features of the individual drugs tested; these characteristics may significantly alter the clinical outcomes [24]. This was highlighted by several reports, where they concluded that an infection with an MDR pathogen alone (without taking into consideration the drugs used for therapy) is not associated with an increased mortality rate, while the use of antibiotics with pronounced toxicities (e.g., colistin in monotherapy or combination therapy) carries a risk of excess mortality [25,26,27]. In the recent years, several novel classification criteria for bacterial resistance were published, with the aim of improving the correlation between resistance data and clinical outcomes: multiple antibiotic resistance (MAR) index [28,29], usual drug resistance (UDR; a definition created by McDonell et al. in 2016, defining resistance which may still be effectively treated with first-line drugs; first used in the context of clinical trials involving novel antibiotics [30,31]) and difficult-to-treat resistance (DTR; a definition created by Kadri et al. in 2018, highlighting resistance to first-line antibiotics with low toxicity; first used in the context of Gram-negative bloodstream infections [24]). 

From the context of human medicine, urinary tract infections (UTIs) are the second most common infections in developed countries, representing an important factor of morbidity and mortality, both among outpatients and hospitalized patients (corresponding to 20%–60% of infections overall) [32,33]. In addition, UTIs are a sizable economic burden for healthcare institutions and national economies: their substantial economic impact is attributable to their pharmacological therapy, hospital costs and lost working days due to recovery; the economic losses caused by UTIs have been estimated to be around three billion US dollars [34]. The most common causes of UTIs in both community and nosocomial settings are Gram-negative bacteria; members of the *Enterobacterales* order are the most prevalent (*E. coli* is the considered the as principal etiological agent, however, the relevance of other members of the order should not be underestimated) causative pathogens, nevertheless, there is an increasing appreciation for the pathogenic role of non-fermenting Gram-negative bacteria (NFGNB; with *Acinetobacter* spp. and *P. aeruginosa* having the highest relevance) in UTIs, especially in patients with predisposing factors for development of complicated UTIs [35,36]. 

Even though the mortality rate associated with UTIs caused by Gram-negative bacteria falls below the mortality of bloodstream infections caused by the same pathogens, due to their high prevalence and constant developments in resistance levels, these infections may be considered as an important target for antibiotic resistance surveillance and stewardship interventions [37]. The epidemiology and patient characteristics of UTIs in our local setting (Albert Szent-Györgyi Clinical Center; Szeged, Hungary) has been extensively characterized previously [20,38,39,40,41]. The aim of the present study was to report on the resistance levels in Gram-negative UTI pathogens in the same healthcare setting retrospectively, using existing and novel classifications of bacterial resistance (which were not previously used in UTIs), over a 10-year study period (2008–2017). In addition, the study aims to introduce novel indicators of resistance, which may be useful in the characterization of resistance in UTIs in the future.

## 2. Materials and Methods 

### 2.1. Study Design, Data Collection

In the present study, microbiological and resistance data was collected retrospectively, corresponding to the time period between 1 January 2008 and 31 December 2017 (10 years) at the Institute of Clinical Microbiology, University of Szeged. The Institute is the primary diagnostic laboratory of the Albert Szent-Györgyi Clinical Center, which is a primary- and tertiary-care teaching hospital in the Southern Great Plain of Hungary (serving as a specialized healthcare center to about 600,000 people, based on the most recent census data) [42]. The collection of the data used in this study was performed manually, in the records of the Institute’s laboratory information system (LIS), corresponding to urine samples positive for Gram-negative pathogens included in this study. Samples where bacterial colony counts were deemed clinically significant (>10^5^ CFU/mL; however, this was subject to interpretation by the senior clinical microbiologists at the time of isolation, based on the information provided on the clinical request forms and international guidelines for the diagnosis of UTIs) and that were positive for the nitrite and leukocyte-esterase tests were included in the data analysis [39]. Only the first isolate per patient was included in the study; however, isolates with different antibiotic-susceptibility patterns from the same patient were considered as different individual isolates. For the purpose of the statistical comparisons, the origin (inpatient vs. outpatient) of the positive samples were also recorded. The study was deemed exempt from ethics review by the Institutional Review Board as no patient data was recorded and data anonymity was maintained.

### 2.2. Identification of Isolates during the Study Period

Ten microliters of each uncentrifuged urine sample was cultured on UriSelect chromogenic agar (Bio-Rad, Berkeley, CA, USA), blood agar (bioMérieux, Marcy-l’Étoile, Lyon, France) and eosin-methylene blue agar (EMB; Bio-Rad, Berkeley, CA, USA) plates with a calibrated loop, according to the manufacturer’s instructions; the plates were incubated at 37 °C for 24–48 h, aerobically [39]. In the first part of the study period (2008–2012), presumptive, biochemical reaction-based methods and VITEK 2 Compact ID/AST (bioMérieux, Marcy-l’Étoile, France) were used for bacterial identification. Starting from 2013, matrix-assisted laser desorption/ionization time-of-flight mass spectrometry (MALDI-TOF MS) was introduced to the workflow of the Institute of Clinical Microbiology. Mass spectrometry was performed by the microFlex LT MALDI Biotyper (Bruker Daltonics, Bremen, Germany) instrument, using the MALDI Biotyper RTC 3.1 software (Bruker Daltonics, Bremen, Germany) and MALDI Biotyper Library 3.1 for the spectrum analysis. The sample preparation procedure, methodology, and the technical details of the MALDI-TOF MS measurements were described elsewhere [39].

### 2.3. Susceptibility Testing of Relevant Isolates

Antimicrobial susceptibility testing for the relevant Gram-negative bacterial isolates were performed based on the methodological recommendations and standards of the European Committee on Antimicrobial Susceptibility Testing (EUCAST) valid at the time of the interpretation; the Kirby–Bauer disk diffusion method (Liofilchem, Abruzzo, Italy), and E-test strips (in case of fosfomycin susceptibility testing; Liofilchem, Abruzzo, Italy) on Mueller–Hinton agar (MHA) plates, in addition, broth microdilution in a cation-adjusted Mueller–Hinton broth (in case of colistin susceptibility testing; MERLIN Diagnostik) was also used. For the verification of discrepant results, the VITEK 2 Compact ID/AST (bioMérieux, Marcy-l’Étoile, France) was used. Susceptibility testing included all relevant antibiotics for the respective bacteria, to allow for their classification into resistance categories based on the criteria detailed in Section 2.4; an antibiotic was not tested and was excluded from the analysis if intrinsic resistance was present, based on the EUCAST Expert Rules on Intrinsic Resistance and Exceptional Phenotypes [43]. To allow for easier data analysis, the bacterial isolates were grouped in six separate groups as follows, based on their similarities in intrinsic resistance: *E. coli*, *Klebsiella* spp., *Citrobacter-Enterobacter-Serratia* spp., *Proteus-Providencia-Morganella* spp., *Pseudomonas aeruginosa* and *Acinetobacter* spp., respectively [20,38,39,40,41]. The interpretation of susceptibility results was performed based on EUCAST breakpoints valid at the time of the interpretation. Intermediate results were grouped with and reported as resistant. The following strains were used as quality control (QC): *S. aureus* ATCC 29213, *E. faecalis* ATCC 29212, *P. mirabilis* ATCC 35659, *E. coli* ATCC 25922, *P. aeruginosa* ATCC 27853, *A. baumannii* ATCC 19606 and *S. maltophilia* ATCC 13637. 

### 2.4. Classification of Isolates into Resistance Categories

During data analysis, respective isolates were classified into various resistance categories. Isolates were considered Wt/susceptible if they were susceptible to all tested antibiotics, excluding those where intrinsic non-susceptibility is present. Isolates were classified in the usual drug resistance (UDR; defined by McDonell et al. [30]) category, if they were resistant to at least one tested antibiotic outside of their realm of intrinsic non-susceptibility. Classification of the isolates as multidrug resistant (MDR; defined as acquired non-susceptibility to at least one agent in three or more antimicrobial categories), extensively drug resistant (XDR; bacterial isolates remain susceptible to only one or two antibiotic categories) and pandrug-resistant (PDR; non-susceptibility to all agents in all antimicrobial categories) was based on the CDC/ECDC recommendations [23]. The designation of difficult-to-treat resistance (DTR; defined by Kadri et al. [24]) was used if an isolate showed resistance to carbapenems (imipenem, meropenem and ertapenem/doripenem), extended-spectrum cephalosporins (the ones relevant for respective pathogens) and fluoroquinolones (ciprofloxacin, levofloxacin and moxifloxacin) [24]. Modified difficult-to-treat resistance (mDTR; introduced in this study, relevant in all bacterial categories, except for *P. aeruginosa*) was used if an isolate showed resistance to extended-spectrum cephalosporins (the ones relevant for respective pathogens) and fluoroquinolones (ciprofloxacin, levofloxacin and moxifloxacin), fosfomycin (for *Enterobacterales* isolates) and sulfamethoxazole-trimethoprim (for *Enterobacterales* and *Acinetobacter* spp.). For *E. coli* only, an additional category (mcDTR; *modified difficult-to-treat resistance in E. coli*) was also introduced, which includes susceptibility data for nitrofurantoin as well. 

As a part of this study, a predictive composite score was also introduced (*pMAR*), which is an augmented version of the multiple antibiotic resistance (MAR) index described previously [28,29], based on the formula (1) below, where *n_MDR_* is the number of MDR isolates in the respective category (e.g., *E. coli*, *Citrobacter-Enterobacter-Serratia*), *AB_MDR_* is the minimum number of antibiotics needed for the respective bacterial group to become MDR, *n_all_* is the number of all isolates in the respective group and *AB_all_* is the number of all antibiotics tested. The *pMAR* value corresponds to the percentage chance (0%–100%) that the isolated pathogen in the present clinical situation will not be treatable by first-line antibiotics, based on local epidemiological characteristics.

(1) $$\mathrm{pMAR}=\frac{n_{MDR}\times AB_{MDR}}{n_{all}\times \;AB_{all}}\times 100\;[\%]$$

### 2.5. Statistical Analyses

Statistical analyses, including the descriptive analysis and statistical tests (Student’s *t*-test and Mann–Whitney U test) were performed with the SPSS software version 24 (IBM SPSS Statistics for Windows 24.0, IBM Corp., Armonk, NY, USA). The normality of variables was tested using Shapiro–Wilk tests. A correlation analysis was also performed with the aim of assessing the temporal nature of changes in the ratio of UDR isolates during the study period; the coefficient of determination (R^2^) was also calculated. These analyses were performed using the Past 3.16 statistical software (Paleontological Museum, University of Oslo; Oslo, Norway). *p* values <0.05 were considered statistically significant.

## 3. Results

During the 10-year study period (1 January 2008–31 December 2017), the study site has received 21,150 urine samples from outpatient clinics and 19,325 samples from inpatient departments that turned out to be positive for a significant urinary pathogen. Out of these isolates, n = 16,240 (76.8%) of outpatient isolates and n = 13,386 (69.3%) of inpatient isolates were Gram-negative bacteria relevant to our analyses (*p* = 0.038). The distribution of isolates is presented in Table 1.

**Figure 1:**
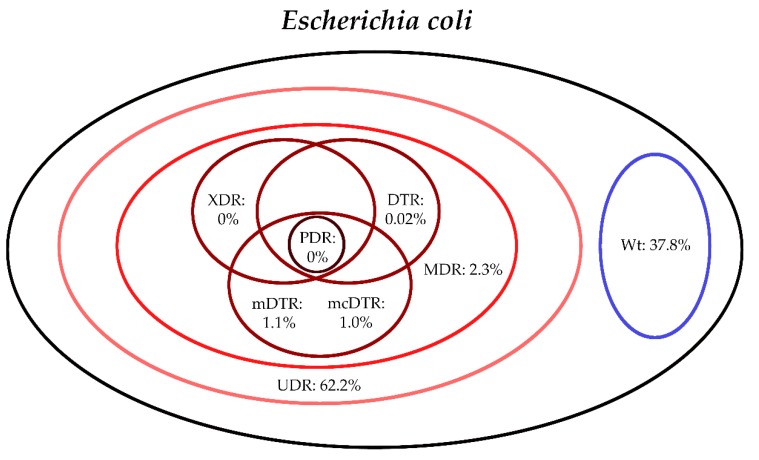
Classification of *E. coli* isolates from UTIs into resistance categories (2008–2017). Variation between inpatient and outpatient isolates is presented in Supplementary Table S1.

**Figure 2:**
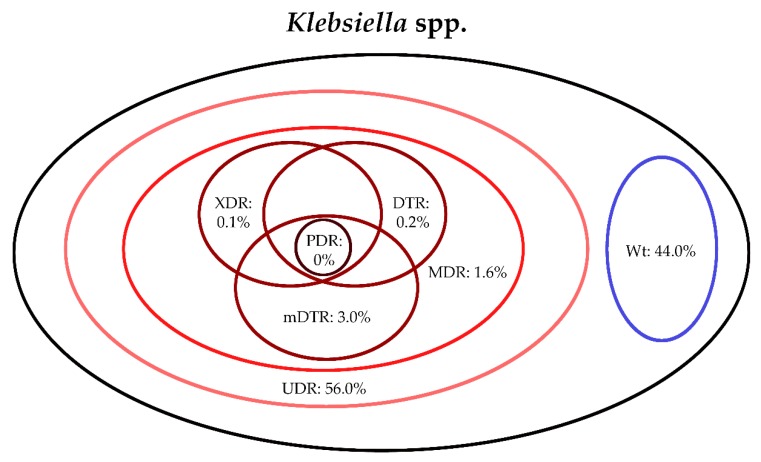
Classification of *Klebsiella* spp. isolates from UTIs into resistance categories (2008–2017). Variation between inpatient and outpatient isolates is presented in Supplementary Table S2.

**Figure 3:**
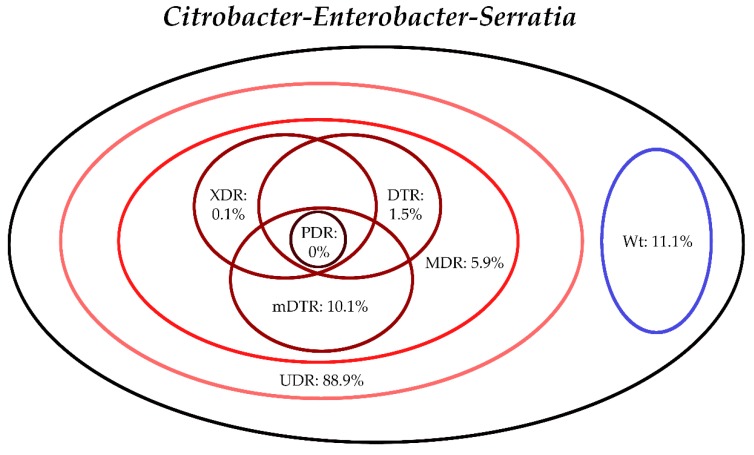
Classification of *Citrobacter-Enterobacter-Serratia* spp. isolates from UTIs into resistance categories (2008–2017). Variation between inpatient and outpatient isolates is presented in Supplementary Table S3.

**Figure 4:**
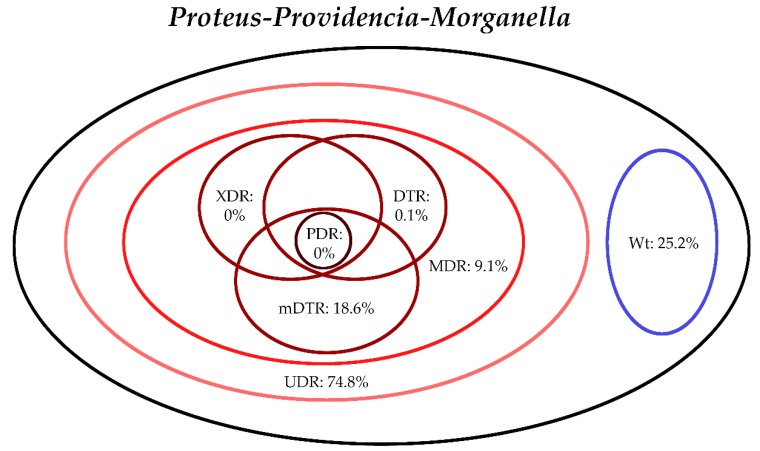
Classification of *Proteus-Providencia-Morganella* spp. isolates from UTIs into resistance categories (2008–2017). Variation between inpatient and outpatient isolates is presented in Supplementary Table S4.

**Figure 5:**
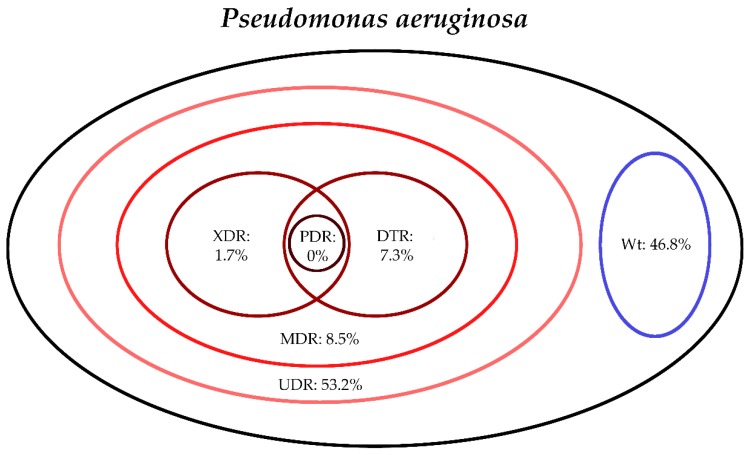
Classification of *Pseudomonas aeruginosa* isolates from UTIs into resistance categories (2008–2017). Variation between inpatient and outpatient isolates is presented in Supplementary Table S5.

**Figure 6:**
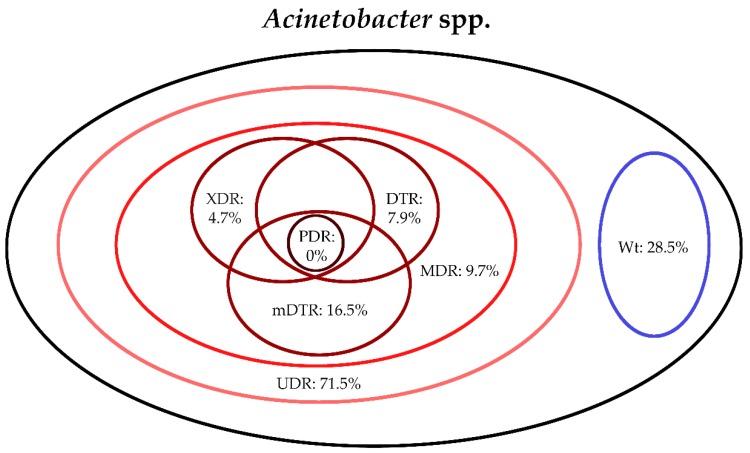
Classification of *Acinetobacter* spp. isolates from UTIs into resistance categories (2008–2017). Variation between inpatient and outpatient isolates is presented in Supplementary Table S6.

**Figure 7:**
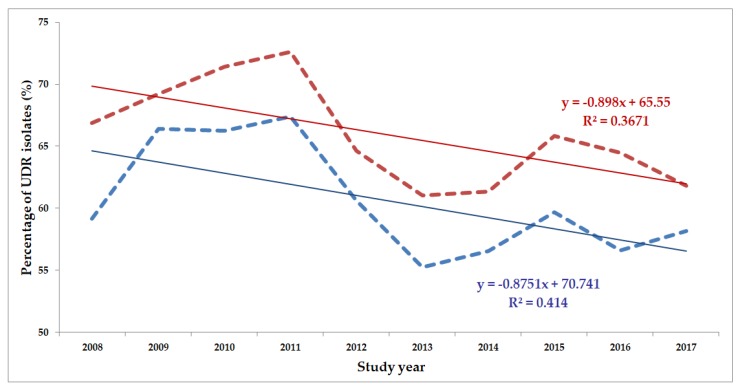
Changing trends of *E. coli* UDR isolates over the 10-year study period (2008–2017). Blue dashed line indicates isolates from outpatients, while red dashed line isolates from inpatients; R^2^: coefficient of determination; R^2^_outpatients_: 0.3671 (36.71%; not significant), R^2^_inpatients_: 0.414 (41.40%; *p* = 0.049).

**Figure 8:**
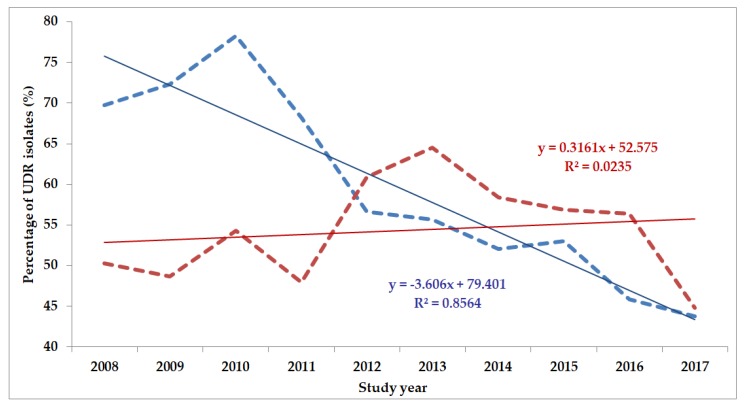
Changing trends of *Klebsiella* spp. UDR isolates over the 10-year study period (2008–2017). blue dashed line indicates isolates from outpatients, while red dashed line isolates from inpatients; R^2^: coefficient of determination. R^2^_outpatients_: 0.8564 (85.64%; p=0.012), R^2^_inpatients_: 0.0235 (2.35%; not significant).

**Figure 9:**
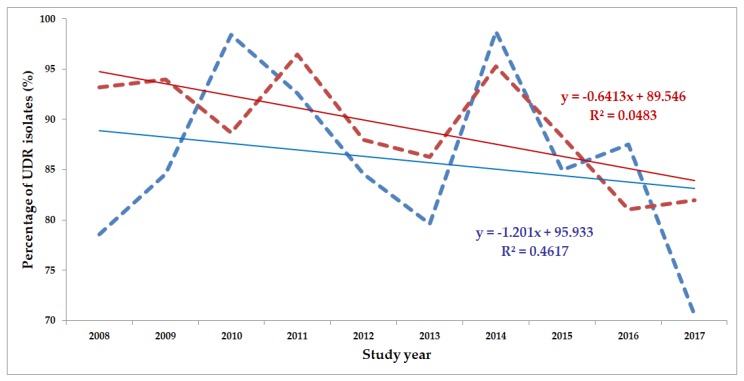
Changing trends of *Citrobacter-Enterobacter-Serratia* UDR isolates over the 10-year study period (2008–2017). Blue dashed line indicates isolates from outpatients, while red dashed line isolates from inpatients; R^2^: coefficient of determination. R^2^_outpatients_: 0.4617 (46.17%; *p* = 0.043), R^2^_inpatients_: 0.0483 (4.83%; not significant).

**Figure 10:**
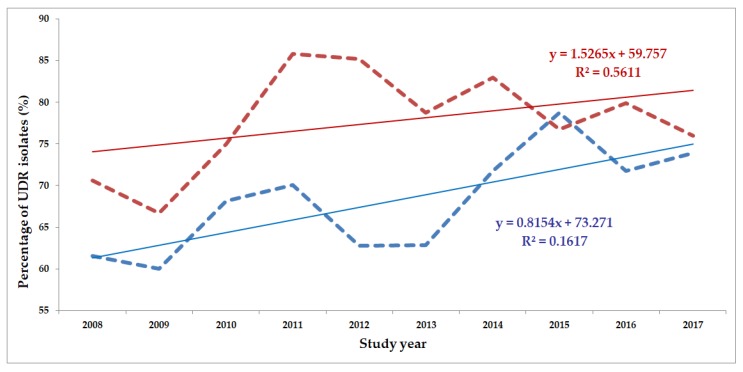
Changing trends of *Proteus-Providencia-Morganella* UDR isolates over the 10-year study period (2008–2017). Blue dashed line indicates isolates from outpatients, while red dashed line isolates from inpatients; R^2^: coefficient of determination. R^2^_outpatients_: 0.1617 (16.17%; not significant), R^2^_inpatients_: 0.5611 (56.11%; *p* = 0.036).

**Figure 11:**
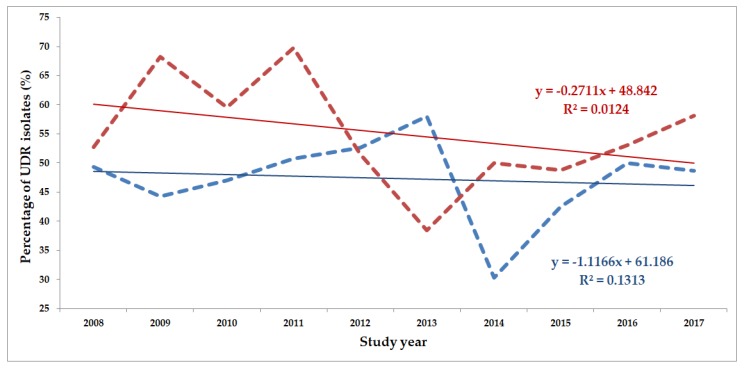
Changing trends of *Pseudomonas aeruginosa* UDR isolates over the 10-year study period (2008–2017). Blue dashed line indicates isolates from outpatients, while red dashed line isolates from inpatients; R^2^: coefficient of determination. R^2^_outpatients_: 0.1313 (13.13%; not significant), R^2^_inpatients_: 0.0124 (1.24%; not significant).

**Figure 12:**
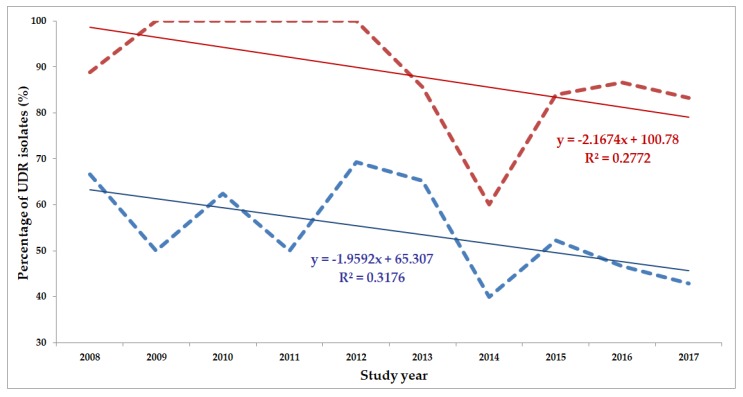
Changing trends of *Acinetobacter* spp. UDR isolates over the 10-year study period (2008–2017). Blue dashed line indicates isolates from outpatients, while red dashed line isolates from inpatients; R^2^: coefficient of determination. R^2^_outpatients_: 0.3176 (31.76%; not significant), R^2^_inpatients_: 0.2772 (27.72%; not significant).

The classification of respective Gram-negative urinary isolates into the resistance categories defined in Section 4 is presented in Figure 1, Figure 2, Figure 3, Figure 4, Figure 5 and Figure 6 (the differences in resistance categories among inpatient and outpatient isolates are presented in Supplementary Tables S1–S6), while the temporal trends associated with the prevalence of UDR isolates over the 10-year study period is presented in Figure 7, Figure 8, Figure 9, Figure 10, Figure 11 and Figure 12, respectively. Out of the *Enterobacterales* isolates groups surveyed, members of the *Citrobacter-Enterobacter-Serratia* (CES) had the highest level of UDR isolates (88.9%; and consequently, the lowest number of wild-type isolates; Figure 3 and Figure 9), while among NFGNB, *Acinetobacter* spp. had high levels of UDR (71.5%; Figure 6 and Figure 12). mDTR levels were highest in the *Proteus-Providencia-Morganella* group (18.6%; Figure 4); the comparison between *Acinetobacter* (16.9%) and *P. aeruginosa* is not possible, as mDTR was not interpreted in that group. MDR levels in *Acinetobacter* spp. and *P. aeruginosa* species were the highest (9.7% and 8.5%, respectively) overall, while among *Enterobacterales,* high levels of MDR were seen in *Proteus-Providencia-Morganella* (9.1%) and *Citrobacter-Enterobacter-Serratia* (5.9%) (Figure 3, Figure 4, Figure 5 and Figure 6.). Levels of DTR were 5-850 times higher in NFGNB than other Gram-negative isolates (7.3% and 16.9% vs. 0.02%–1.5%, respectively). XDR-levels were very low for *E. coli*, *Proteus-Providencia-Morganella*, *Klebsiella* spp., and CES isolates (0%, 0%, 0.1% and 0.1%, respectively; Figure 1, Figure 2, Figure 3 and Figure 4.). Very few isolates were resistant to colistin (n = 0 in *Enterobacterales*, n = 24 for *Acinetobacter* spp. and n = 27 for *P. aeruginosa*) throughout the 10-year study period, however, all of these isolates were susceptible to at least one other agent, therefore, no PDR isolates were detected in any bacterial groups overall (Figure 1, Figure 2, Figure 3, Figure 4, Figure 5 and Figure 6.).

The prevalence of UDR isolates for *E. coli* was lowest in 2013 (Figure 7), for *Klebsiella* spp. in 2017 (Figure 8), for *Citrobacter-Enterobacter-Serratia* in 2017 (Figure 9), for *Proteus-Providencia-Morganella* in 2009 (Figure 10), for *P. aeruginosa* in 2013 and 2014 (Figure 11) and for *Acinetobacter* spp. in 2014 (Figure 12). Varying levels of decreasing tendencies in the levels of UDR isolates were seen over time in almost all isolate groups, except in case of inpatient samples of *Klebsiella* spp. and inpatient/outpatient samples of *Proteus-Providencia-Morganella*, where an increasing tendency was seen. However, statistically significant correlation over time was only observed in inpatient samples of *E. coli* (*p* = 0.049), inpatient samples of *Proteus-Providencia-Morganella* (*p* = 0.036) and outpatient samples of CES (*p* = 0.043).

Based on the previous analysis of susceptibility data, *pMAR* values were calculated, suggesting the percentage chance that the isolates from the bacterial group will be resistant to first-line antibiotics (Table 2); values were ranging between 18.9% and 44.5%, respectively. The highest *pMAR* value was recorded for *Proteus-Providencia-Morganella* isolates (40.3%), while the lowest was seen for *P. aeruginosa* isolates (20.2%) overall.

## 4. Discussion

In the present study, 10-years’ worth of microbiological and resistance data for Gram-negative UTIs in Hungary were assessed, in light of existing and novel resistance classifications [24,28,30]. The surveillance of the etiology and susceptibility patterns in UTIs allow for targeted stewardship interventions in various healthcare facilities [37,44,45,46]. Resistance in Gram-negative bacteria has become one of the main concerns of clinicians (especially since the 2000s) [6]; in addition, plasmid-mediated resistance to carbapenems and colistin in *Enterobacterales* and non-fermenters may lead to widespread dissemination of pathogens with very few treatment options left [17,47,48,49]. Although the severity and mortality rate of UTIs (compared to invasive infections) is less pronounced, the economic burden of drug resistant urinary tract infections is significant, especially if these patients need to be hospitalized [32]. Therefore, the data presented herein may be useful for experts in clinical microbiology, epidemiology and public health, both in Hungary and on the International scene to highlight that growing levels of resistance is a severe issue in the context of UTIs, in outpatients and inpatients alike. Besides the “classical” drug resistance classifications (MDR-XDR-PDR), novel resistance categories found in the literature, and not used before in the context of UTIs (namely UDR and DTR) were also included [24,29,30]. In addition to these, our aim was to further the number of these resistance criteria with additional choices (mDTR, mcDTR and a predictive *pMAR* score) relevant in the classification of UTI pathogens. 

In the report by Kadri et al. [24], the analysis of 46,521 isolates from bloodstream infections (originating from 173 US hospitals) were performed, pertaining to a four-year study period (2009–2013): in their study, n = 471 (1.0%) of isolates were DTR overall, with significant variation among different species (*E. coli*: 0.04%, *Klebsiella* spp.: 1.7%, *Enterobacter* spp.: 0.6%, *P. aeruginosa*: 2.3% and *A. baumannii*: 18.3%). In our study, UDR isolates were the most common in most cases; this classification describes isolates that are not wild-type (i.e., fully susceptible) strains, but their therapy is manageable with standard agents. The case behind DTR (non-susceptibility to all first-line agents, based on Kadri et al. [24]) was that in case of these isolates, resistance is present against all agents with high efficacy, safety and low-toxicity (namely broad-spectrum cephalosporins, fluoroquinolones and carbapenems) and clinicians are forced to use other antibiotics with more disadvantageous or limited pharmacological properties, e.g., aminoglycosides (nephrotoxicity, ototoxicity, neurotoxicity, poor penetration into several anatomical sites), tetracyclines (especially tigecycline in case of drug resistant infections; low serum levels and black-box warning for increased mortality levels), colistin (nephrotoxicity, neurotoxicity, difficulties with formulations and dosing, poor penetration into several anatomical sites) [6,24,50,51]. Nonetheless, resistance to these “reserve” antibiotics should also be monitored to ascertain their clinical utility; for example, based on our previous reports, the levels of aminoglycoside resistance (gentamicin) in UTIs for *E. coli* was between 5% and 15%, 18% and 25% for *Klebsiella* spp., 8% and 25% for CES pathogens, 17% and 20% in *Proteus-Providencia-Morganella*, 31% and 47% for *P. aeruginosa* and 7% and 59% for *Acinetobacter* spp. [20,38,39,40,41], while colistin resistance was <1% in all respective groups, demonstrated in this study. The introduction of mDTR and mcDTR was also indicated, because in many institutions, carbapenem-sparing antimicrobial stewardship regimens are enforced to ensure low levels of resistance against these agents, when they are truly needed [52]. Thus, instead of carbapenems, sulfamethoxazole-trimethoprim and fosfomycin resistance (in addition to nitrofurantoin in case of mcDTR) were taken into consideration, as these are all drugs used as first-line antibiotics in the therapy of urinary tract infections [53,54]. 

Classical resistance classifications (MDR-XDR-PDR) are very useful for epidemiological purposes; however, they may not correlate well with clinical outcomes [24,25,26,27,30,31]. Although several international bodies have published estimates on the excess mortality of MDR infections in Europe [55] and the US [56], these projections have been made based on predictive mathematical models, the relevance of which have been questioned by several reports [57,58]. Likewise, reports published on PDR infections have highlighted that clinical outcomes in their cases was not necessarily associated with the PDR status of the pathogens [59,60]. The category of MDR may be a source of discrepancies; for example, an extended-spectrum β-lactamase-producing and fluoroquinolone-resistant *E. coli* isolate may still be readily treated with fosfomycin. Similarly, ampicillin/sulbactam may still be effective highly resistant *A. baumannii* isolate; both of the mentioned agents are safe and effective alternative [24,39,40]. Another difficulty may stem from the large number of agents (may be as high as 15–17) that need to be tested to verify the XDR/PDR status of an isolate, which is usually not performed in most clinical laboratories [43]; this may lead to the reporting of erroneous results [24]. The latter is further complicated by the introduction of novel antibiotics (e.g., ceftolozane/tazobactam, ceftazidime/avibactam, eravacycline, delafloxacin-meglumine) into the routine susceptibility-testing platforms [16,24]. For the reasons mentioned above, in addition to its clinically-centered philosophy, introduction of DTR (and its modifications detailed in this study) to the bedside and in the clinical practice will definitely lead to substantial benefits in the assessment of the significance of bacterial resistance in human therapeutics [24]. The more frequent application of DTR (and m(c)DTR) by more healthcare-providers and in more published studies (preferably in correlation with clinical data) will lead to its wider recognition.

## Figures and Tables

**Table 1:** Distribution of pathogens from urinary tract infections in our local setting (Albert Szent-Györgyi Clinical Center; Szeged, Hungary) between 2008 and 2017.

Bacterial Isolates	Outpatients	Inpatients
*Citrobacter-Enterobacter-Serratia*	**2.6% (n = 554)**	**3.0% (n = 578)**
*Acinetobacter* spp.	**0.7% (n = 143)**	**0.7% (n = 133)**
*Pseudomonas aeruginosa*	**2.8% (n = 588)**	**5.7% (n = 1096)**
*Proteus-Providencia-Morganella*	**5.0% (n = 1058)**	**7.2% (n = 1392)**
*Klebsiella* spp.	**8.9% (n = 1895)**	**13.4% (n = 2592)**
Gram-positive cocci	**20.7%**	**20.7%**
*Escherichia coli*	**56.8% (n = 12002)**	**42.3% (n = 8173)**
*Candida* spp.	**0.4%**	**6.0%**
Other	2.1%	1.0%

Values in boldface represent isolates included in our analysis.

**Table 2:** *pMAR* values corresponding to respective urinary pathogens in the study period (2008–2017).

Bacterial Group	Outpatients	Inpatients	Overall
*Citrobacter-Enterobacter-Serratia*	37.9%	38.2%	38.1%
*Acinetobacter* spp.	27.1%	44.5%	35.7%
*P. aeruginosa*	18.9%	21.3%	20.2%
*Proteus-Providencia-Morganella*	37.6%	42.6%	40.3%
*Klebsiella* spp.	30.3%	29.5%	29.9%
*E. coli*	25.3%	27.7%	26.1%

## Supplementary Materials

The following are available online at https://www.mdpi.com/2075-1729/10/2/16/s1: Supplementary Table S1. Distribution of Escherichia coli isolates among different resistance categories, 2008–2017; Supplementary Table S2. Distribution of Klebsiella spp. isolates among different resistance categories, 2008–2017; Supplementary Table S3. Distribution of Citrobacter-Enterobacter-Serratia (CES) isolates among different resistance categories, 2008–2017; Supplementary Table S4. Distribution of Proteus-Providencia-Morganella isolates among different resistance categories, 2008–2017; Supplementary Table S5. Distribution of Pseudomonas aeruginosa isolates among different resistance categories, 2008–2017; Supplementary Table S6. Distribution of *Acinetobacter* spp. isolates among different resistance categories, 2008–2017.

## Abbreviations

CDC: Centers for Disease Control and Prevention; CES: *Citrobacter-Enterobacter-Serratia;* CFU: colony-forming units; DTR: difficult-to-treat resistance; ECDC: European Centers for Disease Control and Prevention; EMB: eosine-methylene blue; EUCAST: European Committee on Antimicrobial Susceptibility Testing; ID: identification; LIS: laboratory information system; MALDI-TOF MS: matrix-assisted laser desorption/ionization time-of-flight mass spectrometry; MAR: Multiple antibiotic resistance index; mDTR: modified difficult-to-treat resistance; mcDTR: modified difficult-to-treat resistance for *Escherichia coli*; MDR: multidrug-resistance; MHA: Mueller–Hinton agar; n.s.: not significant; ND4BB: New Drugs 4 Bad Bugs; NFGNB: non-fermenting Gram-negative bacteria; pMAR: a predictive composite score; PDR: pandrug resistance; R^2^: coefficient of determination; QC: quality control; QoL: quality of life; SXT: sulfamethoxazole-trimethoprim; UDR: usual drug resistance; UTI: urinary tract infection; Wt: wild-type; XDR: extensive drug resistance.
