# Supplementary material for: Characterization of Resistance in Gram-Negative Urinary Isolates Using Existing and Novel Indicators of Clinical Relevance: A 10-Year Data Analysis
Source: Life (Basel). 2020 Feb 11;10(2):16. doi: 10.3390/life10020016 (PMC7175163; doi:10.3390/life10020016)
Supplement: Supplementary file 1 [file life-10-00016-s001.pdf]

Supplementary material S1

Characterization of resistance in Gram-negative urinary isolates using existing and novel indicators of clinical relevance: a 10-year data analysis

Márió Gajdács [1]*, Zoltán Bátori[2], Marianna Ábrók[3], Andrea Lázár[3], Katalin Burián[3,4]

[1] Department of Pharmacodynamics and Biopharmacy, Faculty of Pharmacy, University of Szeged; 6720 Szeged, Eötvös utca 6., Hungary

[2] Department of Ecology, Faculty of Sciences, University of Szeged; 6726 Szeged, Közép fasor 52., Hungary; zbatory@gmail.com (Z.B.)

[3] Institute of Clinical Microbiology, Faculty of Medicine, University of Szeged; 6725 Szeged, Semmelweis utca 6., Hungary; abrok.marianna@med.u-szeged.hu (M.A.), lazar.andrea@med.u-szeged.hu (A.L.)

[4] Department of Medical Microbiology and Immunobiology, Faculty of Medicine, University of Szeged; 6720 Szeged, Dóm tér 10., Hungary; burian.katalin@med.u-szeged.hu (K.B.)

* Correspondence: gajdacs.mario@pharm.u-szeged.hu; Tel: +36-62-341-330 (M.G.)

Abbreviation list: CES: *Citrobacter-Enterobacter-Serratia;* **DTR:** difficult-to-treat resistance; **mDTR:** modified difficult-to-treat resistance; **mcDTR:** modified difficult-to-treat resistance for *Escherichia coli*; **MDR:** multidrug-resistance; **n.s.:** not significant; **NFGNB:** non-fermenting Gram-negative bacteria; **PDR:** pandrug resistance; **SXT:** sulfamethoxazole-trimethoprim; **UDR:** usual drug resistance; **Wt:** wild-type; **XDR:** extensive drug resistance

Supplementary Table S1. Distribution of *Escherichia coli* isolates among different resistance categories, 2008-2017

Resistance category	Outpatient isolates	Inpatient isolates	Statistics
**Wt/susceptible**	39.4%	34.1%	n.s.
**UDR**	60.6%	55.9%	n.s.
**mDTR**	0.8%	2.2%	p=0.043
**mcDTR**	0.6%	1.9%	p=0.04
**MDR**	0.2%	3.4%	p=0.018
**DTR**	0.01%	0.01%	n.s.
**XDR**	0%	0%	-
**PDR**	0%	0%	-
**Highest ratio of resistant isolates for:**	Ciprofloxacin	Ciprofloxacin	-

Supplementary material S1

Supplementary Table S2. Distribution of *Klebsiella* spp. isolates among different resistance categories, 2008-2017

Resistance category	Outpatient isolates	Inpatient isolates	Statistics
**Wt/susceptible**	40.4%	45.7%	n.s.
**UDR**	59.6%	54.3%	n.s.
**mDTR**	0.4%	5.9%	p=0.011
**MDR**	0.3%	2.7%	p=0.028
**DTR**	0.2%	0.3%	n.s.
**XDR**	0.1%	0.1%	n.s.
**PDR**	0%	0%	-
**Highest ratio of resistant isolates for:**	Ciprofloxacin	SXT	-

Supplementary Table S3. Distribution of *Citrobacter-Enterobacter-Serratia (CES)* isolates among different resistance categories, 2008-2017

Resistance category	Outpatient isolates	Inpatient isolates	Statistics
**Wt/susceptible**	14.0%	10.1%	n.s.
**UDR**	86.0%	89.9%	n.s.
**mDTR**	5.5%	17.0%	p=0.009
**MDR**	2.0%	8.3%	p=0.016
**DTR**	1.2%	1.9%	n.s.
**XDR**	0%	0.2%	n.s.
**PDR**	0%	0%	-
**Highest ratio of resistant isolates for:**	Ciprofloxacin	SXT	-

Supplementary Table S4. Distribution of *Proteus-Providencia-Morganella* isolates among different resistance categories, 2008-2017

Resistance category	Outpatient isolates	Inpatient isolates	Statistics
**Wt/susceptible**	31.8%	22.2%	p=0.036
**UDR**	68.2%	77.8%	p=0.034
**mDTR**	13.5%	22.9%	p=0.02
**MDR**	7.6%	10.3%	p=0.047
**DTR**	0%	0.2%	n.s.
**XDR**	0%	0%	n.s.
**PDR**	0%	0%	-
**Highest ratio of resistant isolates for:**	SXT	SXT	-

Supplementary material S1

Supplementary Table S5. Distribution of *Pseudomonas aeruginosa* isolates among different resistance categories, 2008-2017

Resistance category	Outpatient isolates	Inpatient isolates	Statistics
**Wt/susceptible**	52.6%	45.0%	n.s.
**UDR**	47.3%	55.0%	n.s.
**MDR**	6.8%	11.4%	p=0.028
**DTR**	5.3%	8.6%	p=0.042
**XDR**	0.8%	2.9%	p=0.049
**PDR**	0%	0%	-
**Highest ratio of resistant isolates**	Ciprofloxacin	Levofloxacin	-

Supplementary Table S6. Distribution of *Acinetobacter* spp. isolates among different resistance categories, 2008-2017

Resistance category	Outpatient isolates	Inpatient isolates	Statistics
**Wt/susceptible**	45.5%	11.1%	p<0.001
**UDR**	54.5%	88.9%	p<0.001
**mDTR**	11.4%	22.6%	p=0.018
**MDR**	7.2%	13.3%	p=0.02
**DTR**	5.0%	11.7%	p=0.024
**XDR**	2.6%	8.8%	p=0.036
**PDR**	0%	0%	-
**Highest ratio of resistant isolates**	SXT	SXT	-
